# Systems immunology: When systems biology meets immunology

**DOI:** 10.3389/fimmu.2025.1630488

**Published:** 2025-09-05

**Authors:** Lucía Alfonso-González, Francisco J. Fernández, M. Cristina Vega

**Affiliations:** ^1^ Abvance Biotech SL, Pharmacokinetics, Pharmacodynamics and Drug Metabolism (PPDM), Madrid, Spain; ^2^ Centro de Investigaciones Biológicas Margarita Salas (CIB-CSIC), Consejo Superior de Investigaciones Científicas, Madrid, Spain

**Keywords:** immunology, mechanistic models, bioinformatics, systems biology, systems immunology, quantitative systems pharmacology, network pharmacology, artificial intelligence

## Abstract

The immune system is an intricate network of cells, proteins, and signaling pathways that coordinate protective responses and, when dysregulated, drive immune−related diseases. Understanding this complexity increasingly relies on systems−based mathematical and computational approaches, which integrate multi−omics data, mechanistic models, and artificial intelligence to reveal the emergent behavior of immune networks. In this mini−review, we discuss the central methodological pillars of systems immunology, including Network Pharmacology, artificial intelligence, and quantitative systems pharmacology. We highlight illustrative applications spanning autoimmune, inflammatory, and infectious diseases, and describe how these methods are used to identify biomarkers, optimize therapies, and guide drug discovery. Finally, we examine current challenges and future directions, including data quality, model validation, and regulatory considerations, which must be addressed to translate systems immunology into clinical impact. This integrated perspective aims to guide both method developers and translational researchers, emphasizing the growing role of computational modeling in next−generation immunology and therapeutic innovation.

## 1 Introduction

The immune system is an extraordinarily complex system, with multiple components interacting to determine the ultimate response (1). Aside from the nervous system, the immune system stands as one of the most intricate and challenging systems in all biology. In quantitative terms, the human brain contains approximately 100 billion neurons, each capable of forming synaptic connections with up to 1,000 other neurons (2, 3). In comparison, the immune system comprises an estimated 1.8 trillion cells and utilizes around 4,000 distinct signaling molecules to coordinate its responses (4, 5). As a result, identifying the key elements and understanding how to effectively modulate specific aspects of the immune response during the treatment of a particular pathology can be challenging. Mathematical and computational modeling provide valuable insights into the relative importance of immunological components and their alterations under various conditions (6). These are particularly useful for studying the pathological mechanisms underlying immune-related diseases and evaluating the mechanisms of action of therapeutic agents. By incorporating pharmacokinetic and pharmacodynamic (PK/PD) considerations, mathematical models can facilitate the optimization of new treatment strategies discovery, enabling more efficient comparisons of therapies targeting different immune pathways or exhibiting diverse PK/PD profiles.

From the perspective of immunology, the mammalian immune system is a complex and highly specialized network of molecules, cells, tissues, and organs that recognize, respond to, and eliminate pathogenic organisms and abnormal self-components while maintaining tolerance to self-antigens. It operates through two complementary arms: the innate immune system, which provides immediate, nonspecific defense, and the adaptive immune system, characterized by antigen-specific responses, immunological memory, and clonal expansion. The system integrates humoral and cellular components, mediated primarily by leukocytes (e.g., lymphocytes, macrophages, dendritic cells) and secreted factors (e.g., cytokines, antibodies), to maintain homeostasis and prevent disease. Immunology traditionally describes the immune system in structural and functional terms, emphasizing the components that exist and their respective functions.

From the viewpoint of systems biology, the mammalian immune system is understood as a dynamic, multiscale, and adaptive network composed of heterogeneous cellular and molecular entities interacting through complex signaling pathways, feedback loops, and regulatory circuits. It exhibits emergent properties such as robustness, plasticity, memory, and self-organization, arising from local interactions and global system-level behaviors. In systems biology, the immune system is modeled as an open system interacting with internal (e.g., microbiota, neoplastic cells) and external (e.g., pathogens, environmental cues) agents, with a focus on quantifying and simulating the spatiotemporal dynamics of immune responses through computational and mathematical modeling. Thereby, systems biology conceptualizes the immune system as an interconnected and evolving network, emphasizing how interactions and systemic properties emerge from component interactions.

The foundation of systems immunology as a distinct field of inquiry goes back to the realization of the need for careful observation and rigorous analysis to understand the extraordinary complexity of the mammalian immune system. The modern concept of this field can be traced back to the publication of three landmark articles around 2008-2009: an editorial titled “A prescription for human immunology” by the immunologist Mark M. Davis (7) and the pioneering studies by the groups of Sékaly (8) and Pulendran (9) on the immune response to Yellow Fever vaccine using gene expression arrays and other large-scale biological data. These seminal efforts highlighted a critical need and a way forward: the advance of human immunology required new approaches because most experimental strategies used in mice were not feasible in humans, and the recommendation to use quantitative metrics and informatics for data mining, analysis, modeling, and, eventually, prediction to aid basic understanding and therapeutic efforts.

In this context, it becomes clear that studying the immune system from a systems biology perspective is essential. In this mini-review, we summarize systems immunology as an innovative and exciting field and discuss how it integrates with and enhances traditional research methods by combining omics techniques with advanced mathematical modeling. The main goal is to accurately predict the immune system as a whole and apply this knowledge to develop more effective treatments for immune-inflammatory conditions. Our primary audience includes potential users of systems biology and computational approaches, such as bioinformaticians, computational biologists, and interested immunologists who may want to use these methods to study immune function and dysfunction. General immunologists and clinicians form a secondary audience, as systems immunology ultimately seeks to generate insights that improve understanding and treatment of immune−related disorders in real-world clinical settings.

The review is structured to serve both audiences. We first introduce the methodological and systems−level approaches that form the foundation of systems immunology, aimed at researchers and computational scientists seeking to apply these tools. Subsequent sections illustrate these methods with concrete examples in immune−related diseases, drug discovery, and therapeutic development, demonstrating how systems−level insights can inform real−world translational applications. By progressing from conceptual foundations to clinical relevance, the review highlights both the analytical depth needed by method developers and the practical insights sought by immunologists and clinicians.

## 2 From complexity to systems immunology

Systems Biology entails the integration of quantitative molecular measurements with computational modeling of molecular systems at the organism, tissue, or cellular level. By integrating all components within the system under investigation, the aim is to gain a comprehensive understanding of the broader biological context (10).

**Figure 1:**
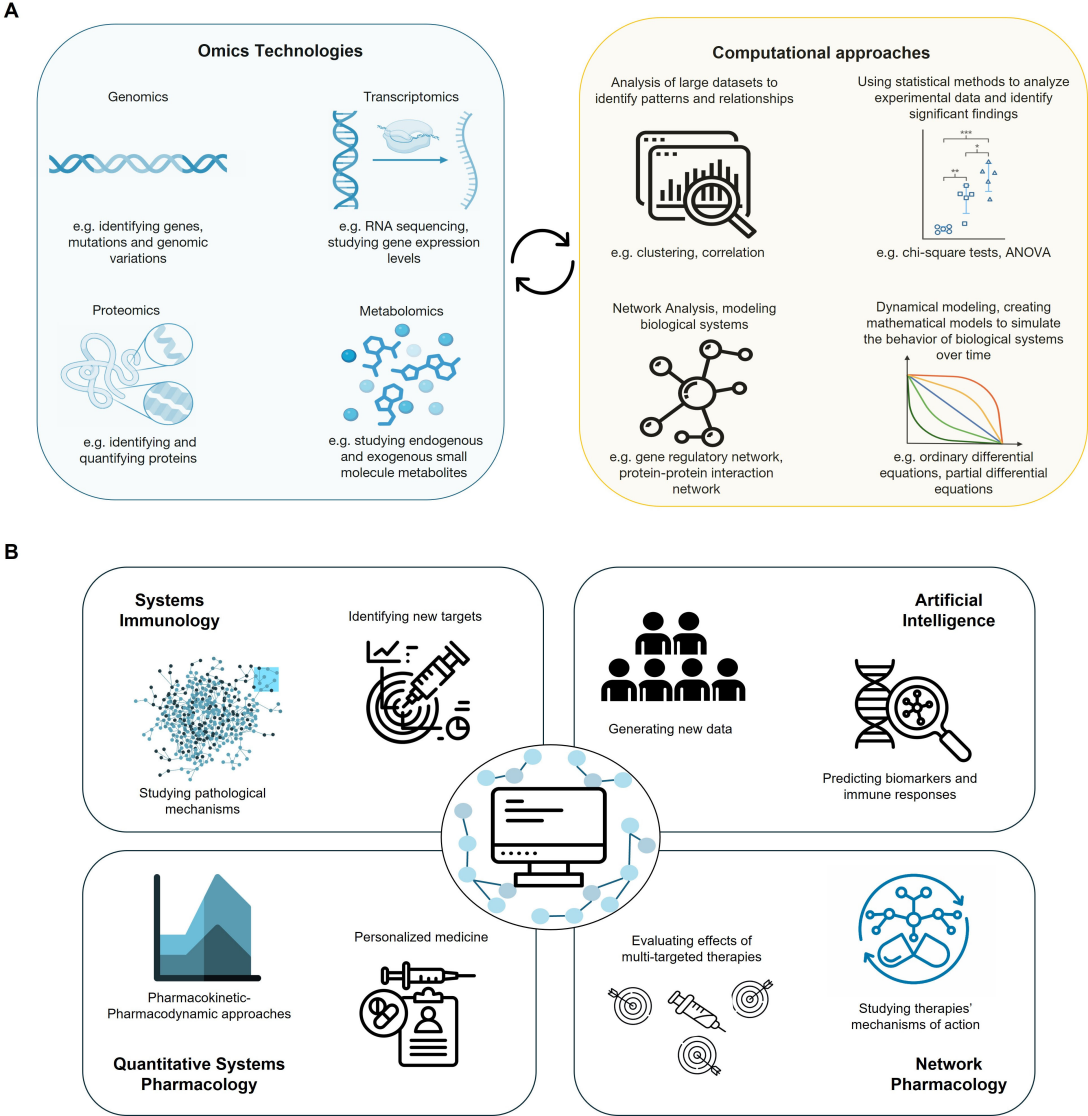
Computational modeling in Immunology. **(A)** Integration of various omics methodologies widely used in immunology research into a coherent systems immunology field through the integration by computational and mathematical tools. Top panel - Omics technologies: Representative tools for generating high−dimensional immune datasets, including transcriptomics (e.g., RNA−seq), proteomics (e.g., single-cell CyTOF), and metabolomics platforms. Bottom panel - Computational approaches: Examples of methods for analyzing and modeling immune complexity, including statistical modeling, ordinary differential equation (ODE) models for dynamical simulations, and network inference algorithms. Together, these methods support the integration of immune data and mechanistic modeling to uncover disease mechanisms and inform therapy development. **(B)** Examples illustrating the applications of systems immunology, artificial intelligence, network pharmacology, and quantitative systems pharmacology, and their translational applications. The diagram illustrates how systems immunology, AI/machine learning, network pharmacology, and QSP modeling intersect to generate actionable insights. Examples of intersections - AI + QSP: AI−driven biomarker discovery supports model parameterization and therapy response prediction. Network + Mechanistic Models: Network−derived signaling modules integrated into ODE−based QSP models for immune pathway simulation. Together, these intersections facilitate drug discovery, biomarker identification, and personalized therapy design in immune−mediated diseases.

Systems Biology is especially relevant in its application to immunology, giving rise to Systems Immunology (**Figure 1A**). The immune system is particularly complex, comprising numerous cells that exhibit diverse activation states and interact with one another, as well as with cytokines and chemokines. Research through systems immunology aims to understand the interactions between various components, the contribution of each element to the system’s response, and ultimately, to predict the dynamics and response to specific phenomena affecting the system (11, 12). Thus, computational modeling can provide valuable insights into the relative importance of different immune components, the influence of other elements on them, and how their relationships may change under various conditions. In this way, a map of the system’s integrated functioning is developed, enabling the identification of potential targets for the clinical modulation of the immune response by generating informed hypotheses that can be contrasted with experimental analyses (6).

System-based approaches have focused almost entirely on data-driven approaches based on ever-expanding ‘omics’ data sets (e.g., transcriptomics, proteomics, metabolomics) (**Figure 1A**). Single-cell technologies, including scRNA-seq, CyTOF, and single-cell ATAC-seq, are transforming systems immunology by revealing rare cell states and resolving heterogeneity that bulk omics overlook. These datasets provide high−dimensional inputs for data analysis, enabling cell−state classification, trajectory inference, and the parameterization of mechanistic models with unprecedented biological resolution. Clinically, single−cell analyses are beginning to inform patient stratification and biomarker discovery, strengthening the translational bridge from data to therapy. Computational models used to analyze these datasets can range from simple curve fitting or regression modeling to artificial intelligence methods, which are becoming increasingly prevalent with the growing availability of rich data sets (6).

Mechanistic models are quantitative representations of biological systems that describe how their components interact (**Figure 1A**). The construction of mechanistic models is determined and limited by the knowledge of the system under consideration. Their validity is based on their ability to predict one or more known behaviors of the systems and previously unobserved behaviors. Analogous to experimental studies on biological systems, in silico experiments on mechanistic models enable the generation of novel hypotheses that may not emerge from empirical data alone and that would have otherwise been difficult to formulate. Although these tools have had a relatively minor impact on immunology so far, they have been widely used in other areas of biology. In cardiovascular biology, for example, multi-scale computational models based on the Hodgkin-Huxley mechanistic model have a predictive value for human toxicology that surpasses that of experimental rabbit models and are accepted by the Food and Drug Administration (FDA) as appropriate methodologies for understanding therapy-induced cardiotoxicities (13). One of the limitations of mechanistic models is that they necessitate a thorough understanding of the system being studied, even though unknown parameters are usually addressed through assumptions or by fitting experimental data. The construction of these models is also slow and laborious, although once implemented, they can carry out hundreds of virtual tests in a short time (6, 14).

## 3 Artificial intelligence for immune system analysis

Artificial intelligence (AI) refers to a class of computational systems capable of displaying intelligent behavior by analyzing their environment and making decisions, with some degree of autonomy, to achieve specific goals. This broad field encompasses techniques ranging from classical machine learning algorithms, including support vector machines and single-layer neural networks, to more advanced approaches such as deep learning (15). Machine learning (ML) techniques involve the development of algorithms that learn from data, identify patterns, and make predictions or decisions with minimal human intervention. Deep learning is one of the most advanced and complex machine learning techniques. It utilizes models with multiple layers, such as convolutional or recurrent neural networks, which allow the extraction of high-level features and patterns from large, complex datasets (16).

The development and performance of AI models in immunology are critically dependent on the size, diversity, and quality of the datasets used. Robust, reliable models require high-quality annotations, representative biological variation, and carefully curated metadata (17). Ideally, artificial intelligence models should exhibit interpretability, clinical relevance, versatility, and reliability. Moreover, they must address ethical considerations, including data privacy, informed consent, and algorithmic bias, remaining sensitive to the specific contexts in which they are deployed (18, 19).

Applications of AI in immunology include supporting the discovery of novel biological pathways, predicting biomarkers and immune responses, and generating new data through generative AI techniques. An example of new pathway discovery is the work by Sparks et al., who developed ML models using multi-omics data (transcriptomics, proteomics, and immune cell profiling) to improve diagnostics in autoimmune and inflammatory diseases, as well as to predict vaccine responses (20).

Progress has been made in the prediction of biomarkers and immune responses, including the development of disease-specific AI models in asthma (21), cancer (22, 23), and vaccination (24, 25), often improving upon conventional statistical approaches in both performance and scalability.

Single-cell omics deserve particular attention in this context, as they enable the integration of diverse molecular dimensions within individual cells, allowing for precise discrimination of developmental states and cell types. This high-resolution data serves as an ideal foundation for training artificial intelligence models (26, 27). A clear example is the machine learning approach developed by Xu et al., for the identification of neutrophil clusters and novel biomarkers relevant to sepsis (28).

Generative AI, while still in its early stages in immunology, holds significant promise. These models, trained on large-scale datasets, can generate novel data for hypothesis generation, virtual experimentation, and simulation of biological scenarios (29). Potential applications in immunology include drug discovery, precision immunotherapy, and in silico clinical trial design (18, 30).

Together, these AI approaches not only advance mechanistic understanding of immune processes but also support translational applications, including biomarker discovery, patient stratification, and the design of personalized immunotherapies.

## 4 Network pharmacology in immune pathways

There is a growing interest in integrating pharmacology and Systems Biology, as evidenced by an increasing number of publications since 2020. The collaboration between these two fields has led to the generation of a new discipline, Network Pharmacology (31). Network pharmacology extends systems immunology by integrating multi−omics data, drug-target interactions, and disease networks to reveal how therapeutic interventions perturb complex immune systems. Unlike traditional single-target approaches, network pharmacology evaluates sets of molecules and pathways, acknowledging that immune-mediated diseases often arise from multi-node dysregulation.

Typical network pharmacology workflows begin with assembling a disease−associated network using genomic, transcriptomic, and proteomic datasets. Computational methods such as network topology analysis, community detection, and centrality scoring are then used to identify critical nodes and subnetworks that can serve as potential drug targets. Drug-target networks can be superimposed to evaluate multi−target strategies or predict off−target effects, enabling rational polypharmacology. Network Pharmacology is an effective tool for analyzing complex interactions, identifying novel therapeutic targets, investigating the underlying causes of treatment inefficacy, and assessing toxic and beneficial interactions among different components.

The use of Network Pharmacology techniques is increasingly popular for understanding the mechanisms of multi-target drugs in treating complex and multifactorial diseases, including autoimmune disorders and conditions such as ulcerative colitis (32–35), vitiligo (36), asthma (37), and rheumatoid and gouty arthritis (38, 39). Recent applications include mapping cytokine networks to prioritize multi-cytokine blockade strategies in rheumatoid arthritis (RA) (40), as well as analyzing immune cell-drug interaction networks to mitigate hyperinflammatory responses in COVID-19 (41).

By combining pathway−level insights with drug-target mapping, Network Pharmacology serves as a bridge from multi−omics discovery to therapy prioritization. This approach supports both mechanism discovery, by highlighting critical signaling modules and their redundancy or compensation, and drug repurposing and polypharmacology, enabling rapid translation of network−informed hypotheses to preclinical or early clinical testing.

## 5 Quantitative systems pharmacology in immunology and therapy development

Pharmacology researchers use different mathematical approaches to build integrated pharmacokinetics/pharmacodynamics (PK/PD) models for drug action in a biological system. These models rely heavily on obtaining experimental data on the drug and associated biological responses collected over time and at various doses. PK/PD models, similar to mechanistic models of Systems Biology, are integrated using ordinary differential equations (ODEs) to describe the processes of absorption, distribution, metabolism, and excretion (ADME) of the drug in the organism and its binding to the target (42).

Although PK/PD models and simulations have been a part of clinical development since the 1980s, model-based drug development is a much more recent phenomenon, increasingly advocated by industry, academia, and, especially, regulatory agencies, including International Conference Harmonization (ICH), U.S. Food and Drug Administration (FDA), European Medicines Agency (EMA), Japanese Pharmaceuticals and Medical Devices Agency (PMDA), and China’s National Medical Products Administration (NMPA) (43–48).

Quantitative Systems Pharmacology (QSP) approaches are based on the principles of Systems Biology and pharmacology (**Figure 1B**) to generate mechanistic models of physiology in health and disease along with PK/PD models of drugs to predict their effects on the system as a whole, providing a framework for translational research that quantitatively links pharmacological targets, physiological pathways, and, ultimately, integrated disease systems (49). QSP models have a growing impact on model-informed drug discovery and development (50). Its usefulness is recognized at all stages of drug development, from its initial discovery to its growth in later stages. It is also helpful during the management of the drug in clinical practice and serves as a support during its regulatory submission (50, 51).

In drug discovery, QSP models can identify potential targets for new treatments, enabling the evaluation of molecules with different pharmacokinetic properties. During clinical development, QSP models help reduce costs in clinical trials by improving project selection and progression, facilitating an assessment of their relative risks, and avoiding approaches with a low probability of success (42, 52). QSP models are also valuable in patient management in clinical practice, as they allow model parameters to be customized based on the genetic and epigenetic profiles of individual patients, thus providing a pathway for personalized medicine (42).

Despite all these advantages, the potential and the growing number of models, the in-depth interaction between mechanistic modeling and experimental and clinical research, particularly in immunology, remains a relatively uncommon practice. Some notable QSP models in the field of immunology include approaches for identifying new therapeutic strategies and determining the mechanism of action of certain drugs and its potential application in immune-oncology (53–55), sepsis (56), autoimmune diseases such as Crohn’s disease (57, 58), systemic lupus erythematosus (59), and RA (60, 61), as well as therapies targeting the complement system (62–64), and even during bacterial (65) and viral infections (66).

Additionally, approaches continue to be novel, and the methodology is still under discussion. The community and regulators have not agreed on precise QSP model development guidelines (14). Use cases must be carefully selected to ensure the models’ valid application. For QSP models to receive greater acceptance in clinical practice, the values assigned to parameters and relevant interactions should be thoroughly examined through sensitivity analysis and recognized as plausible by both immunologists and clinical researchers.

By integrating immunological mechanisms with pharmacokinetics and pharmacodynamics, QSP models have become a critical link between preclinical studies and clinical decision−making, guiding dose selection, trial design, and the development of immune−targeted therapies.

## 6 Challenges and future directions

The realization of systems immunology’s potential must address challenges and limitations. Some limitations arise from the inherent variability and heterogeneity of biological samples, as well as the complexity of the omics techniques required to analyze complex samples. Standardization of sampling protocols, analytical methods, and, if ethically permissible, unrestricted data sharing should all contribute to producing reproducible and trustworthy experimental results and widely accepted conclusions.

Other limitations involve the computational models used to analyze and interpret systems-level properties. The main restriction is the strict need for high-quality, abundant data to train AI models. Because immunology datasets are often high−dimensional but limited in size, AI models face a substantial risk of overfitting, which can compromise generalizability. Moreover, reproducibility remains challenging without standardized pipelines and open benchmarking datasets, underscoring the need for transparent and well−curated data.

Large, multiscale QSP models encounter scalability challenges due to increased dimensionality and computational demands, which restrict their application. Validating these models with independent datasets is crucial for ensuring reliability and gaining regulatory approval, particularly when translating insights from model organisms to humans. Achieving comprehensive and accurate validation for both animal and human predictions will require additional effort in the future.

A final reflection concerns the regulatory challenges faced by outcomes from systems immunology. Medical regulatory agencies like the FDA and the EMA have indicated they welcome computational mechanistic modeling of new drugs’ effects, even though regulatory adoption of systems−based models is progressing cautiously. Examples include cardiovascular QSP models and PK simulations that have informed dose selection and safety assessment in FDA and EMA submissions. Despite this increasing regulatory interest, the clinical adoption of systems-based methods remains limited by the lack of standardized modeling practices and reproducible pipelines. These gaps, along with the need for rigorous independent validation, continue to slow the broader translation of AI and QSP approaches into regulatory decision-making. Understandably, any mistakes in the quantitative modeling of the immune system or its components with consequences for clinical trials could predictably cause increased regulatory concerns and hurdles.

It is hopeful that the systems immunology community recognizes these limitations and is actively working to find solutions. Much work has been done by omics researchers to address key experimental issues, such as reproducibility. Computational models are also becoming more accurate, and their predictions more precise. Given the rapid pace of new discoveries and the publication of innovative bioinformatics tools in systems immunology, the future looks promising for this exciting field.

## 7 Conclusions

Mathematical modeling in systems biology provides a powerful framework to simulate and analyze complex interactions among multiple biological components. By integrating these elements into understandable networks, such models allow the study of biological systems whose complexity might otherwise hide key functional relationships. This is especially important in immunology, where the dynamic and layered nature of the immune response creates significant challenges for traditional analysis methods. Immunological diseases and disorders are difficult because of the complexity of the immune response. Combining systems biology with immunology opens new possibilities for uncovering the molecular roots of immunological diseases. This integration helps identify critical regulatory nodes and signaling pathways, offering new insights into disease development and treatment (**Table 1**). Artificial intelligence further enhances this potential by enabling the analysis of high-dimensional immunological data, supporting biomarker discovery, disease outcome prediction, and the development of precision therapies. However, the large amount of data needed to build reliable AI models highlights the importance of a strong, accessible, standardized, and high-quality data infrastructure. At the same time, pharmacology-based modeling approaches, including Network Pharmacology and QSP, provide mechanistic insights into drug action and immune modulation. These approaches require careful selection of databases and algorithms, as well as high-quality research data (67). These models provide a computational platform for comparing, improving, and optimizing treatment strategies, thereby aiding decision-making throughout all phases of drug development and clinical care. Overall, the integration of systems biology, artificial intelligence, and pharmacological modeling enhances our ability to understand, predict, and control immune function. Further progress in these integrated methods within immunology will be crucial to unlock their full potential for research and clinical use.

**Table 1:** Systems immunology stages.

**1. Identify the most prominent components in a biological system and/or phenomenon** • **Identification of the biological system and its major components.** This involves defining the scope of the model, including the relevant cells, genes, proteins, metabolites, and other molecules. • **Search for experimental data**, including gene expression, protein and metabolite concentrations, and other relevant components. • **Analyze the collected data**. Identify relationships and trends that may affect the model.
**2. Looking for interacting components within a phenomenon** • **Collects information on model interactions and experimental data describing the kinetics of these interactions.** Sometimes it is necessary to estimate unknown parameters based on experimental data.
**3. Model formulation, predictive simulation and analysis within a phenomenon and/or system-wide** • **Choose the appropriate modeling approach.** This can include using different modeling techniques such as ordinary differential equations (ODEs), constraints-based models and agent-based models. • **Build the model and perform simulations.** • **Analyze the results and validate the model**. Analyze the behavior of the model, and compare the results obtained with experimental data and other approaches. • **Refine the model**. Modify the model based on the results of the validation, this can lead to the variation of the parameters or the addition of new components. • **Iterate the process.** The acquisition of data and model formulation, simulation and analysis are often repeated iteratively to refine the model and deepen understanding of the system.

Inspired by Davis (12), these stages have been tailored to the development of QSP models in systems immunology, using a Boolean network analysis used in sepsis (56), Entelos^®^ Rheumatoid Arthritis PhysioLab^®^ platform (61), and C-model, a QSP model centered on the complement system (64), as examples.

Panel titles corresponding to the main modeling stages are shown in boldface.
